# High-Grade Serous Carcinoma Resulting From Rectal Endometriosis and Complicated With Ovarian Cancer

**DOI:** 10.3389/fonc.2019.01252

**Published:** 2019-11-20

**Authors:** Li Song, Aiyan Xing, Qiuju Li, Guoyun Wang

**Affiliations:** ^1^Department of Obstetrics and Gynecology, Qilu Hospital, Shandong University, Jinan, China; ^2^Department of Pathology, Qilu Hospital, Shandong University, Jinan, China

**Keywords:** endometriosis-associated malignancy, rectal endometriosis, high-grade serous carcinoma, simultaneous primary carcinoma, ovarian cancer

## Abstract

Endometriosis is one of the most common benign gynecological diseases. It shows similar attributes to those of some fatal tumors if it is becomes malignant. These attributes include invasion, implantation, and recurrence. Epidemiological, clinicopathological, molecular biological, and genetic evidence suggest that malignancy of endometriosis, referred to as endometriosis-associated malignancy (EAM), is histologically closely related to endometriosis. Atypical endometriosis, which usually causes EAM, is considered a transitional condition from benign endometriosis to cancer. Approximately 80% of EAMs occur in the ovary and are known as endometriosis-associated ovarian cancer (EAOC). However, extragonadal endometriosis is not common, and no earlier study reported an association between malignant transformation of rectal endometriosis and high-grade serous cancer. We report a rare case of high-grade serous carcinoma resulting from rectal endometriosis and complicated with ovarian cancer. A 63-year-old Chinese woman was admitted with a complaint of abdominal distension. We diagnosed the patient with ovarian carcinoma and decided on elective cytoreductive surgery as treatment for the patient. During the surgery, we found a solid mass of diameter 12 cm in the anterior rectal wall containing sticky brown fluid. Postoperative histopathological examination revealed high-grade serous carcinoma resulting from rectal endometriosis and complicated with ovarian cancer. The patient postoperatively received 6 cycles of chemotherapy consisting of carboplatin and paclitaxel and was followed up for 1 year with no recurrence of the condition.

## Background

Endometriosis is a common and complex disease with an incidence of about 6–10% and major manifestations of dysmenorrhea or infertility. The severity of its symptoms is not necessarily related to the clinical stage of the disease, and its degree of progression cannot be predicted. Earlier studies suggested that endometriosis increases the risk of some malignant tumors, 80% of which occur in the ovary. However, extragonadal endometriosis accounts for 5.7% of all malignant transformations of endometriosis, 4.3% of which occur in the rectovaginal septum, and another 4.3% in the colorectum. We report a case of high-grade serous carcinoma in extragonadal sites of endometriosis.

## Case Presentation

**Figure 1:**
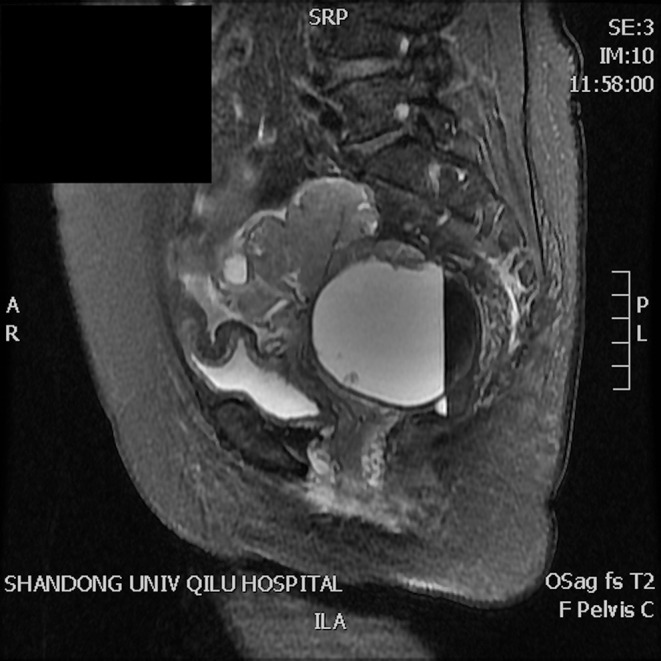
Magnetic resonance imaging revealing solid, cystic, irregular, and space-occupying lesions in the left adnexal area and before the rectum, presenting long T2 signals and equal short T1 signals. The solid part of the diffusion-weighted imaging lesion shows high signal intensity with unclear boundaries, the cystic part shows the liquid level, enhanced scanning of the solid part reveals obvious heterogeneous enhancement, and the boundary between the lesion and adjacent structures is unclear.

**Figure 2:**
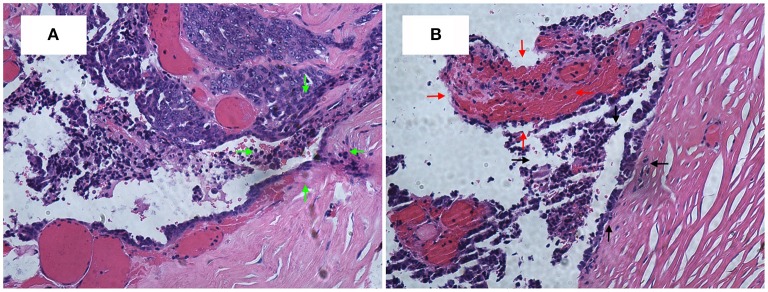
**(A)** High-grade serous carcinoma is contiguous with endometriosis, infiltrating throughout the sub-serosa. H&E, ×200. **(B)** The black arrow area is the endometrial gland, and the red arrow area is the stroma.

**Figure 3:**
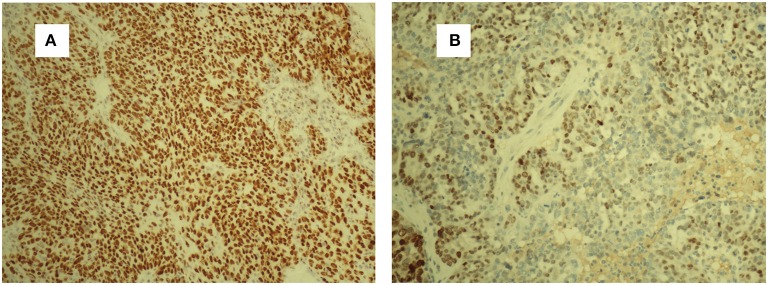
Strong WT-1-positive staining of tumor cells. WT-1 **(A)**. ER-positive staining of the endometrial glands and stromal cells **(B)**. ER, estrogen receptor.

A 63-year-old woman was hospitalized for abdominal distension. The levels of carcinoembryonic antigen and carbohydrate antigen (CA) 19-9 showed no elevation before surgery, and the CA125 level was 258.32 U/ml. Ultrasonography showed that the left posterior aspect of the uterus increased in size owing to the presence of a solid cystic mass 12 cm in diameter, of irregular shape, and with unclear boundaries. The solid part presented circulation signals, whereas the cystic part showed poor sound penetration with fine dot echo. The left ovary was enlarged, which implied that space-occupying lesions were present. Other findings included adenomyoma and adenomyosis, mass in the left posterior aspect of the uterus, and ascites. We performed rectovaginal examination of the patient before surgery, and the anterior rectal wall had a cystic mass of about 10 cm, which was convex toward the rectum. Pelvic magnetic resonance imaging revealed solid, cystic, irregular, and space-occupying lesions in the left adnexal area and before the rectum, presenting long T2 signals and equal short T1 signals. The thickness of rectal wall was increased. The solid part of the diffusion-weighted imaging lesion showed high signal intensity with unclear boundaries, whereas the cystic part showed the fluid level, and enhanced scanning of the solid part revealed obvious heterogeneous enhancement, and the borders between the lesion and adjacent structures were unclear (Figure 1). The patient was diagnosed with ovarian carcinoma on the basis of the ultrasound results and was scheduled for cytoreductive surgery. During the surgery, ~1,000 ml of light bloody fluid was found in the pelvic cavity, and the left ovary was enlarged by the presence of a 5 × 4-cm solid cystic mass with a surface resembling a cauliflower. The omentum is thickened, and the surface is smooth. No significant abnormalities were found in the omentum and lymph nodes. Smooth solid cystic mass of 12 cm in diameter was found in the anterior rectal wall in the pelvis and partially adhered to the left peritoneum. The part of the mass that adhered to the left peritoneum was resected on suspicion of peritoneal invasion. Thereafter, we carefully separated the mass along the surface of the rectum and completely removed the mass. In the process, the sticky brown liquid flowed out, and the inner wall of the tumor was seen to have brittle papillary protrusions. After removing the rectal mass, we sutured the serosal layer of the rectum. Examination of frozen sections confirmed adenocarcinoma, but it did not reveal peritoneal invasion. Cytoreductive surgery was then performed and included routine hysterectomy and bilateral salpingo-oophorectomy, lymph node dissection, appendix dissection, and omental dissection. Histopathological examination revealed high-grade serous carcinoma resulting from rectal endometriosis and complicated with bilateral high-grade serous ovarian carcinoma, but it showed no lymph node metastasis. Photomicrographs of the sampled cyst wall showed fibrous connective tissue, hemorrhagic denaturation, and hemosiderin deposition. Contiguous with endometriosis, the high-grade serous carcinoma infiltrated throughout the sub-serosa, as shown in Figures 2A,B. The immunohistological examination indicated that WT-1 was positively stained and that the rectal tumors near endometrial glands were positive to estrogen receptor (ER) staining (Figures 3A,B). A diagnosis of high-grade serous carcinoma resulting from rectal endometriosis and complicated with ovarian cancer was finally made.

## Discussion

The biological behavior of malignant tumors is typically characterized by growth and invasion, distant metastasis, and frequent recurrence. Endometriosis is a common gynecological disease. Histologically, endometriosis-associated malignancy (EAM) is closely related to endometriosis (1). It was reported that the malignant transformation rate of endometriosis is 0.7–1.0%, of which 80% occur in the ovary. However, extragonadal endometriosis accounts for 5.7%, of which 4.3% occur in the rectovaginal septum and another 4.3% in the colorectum (2). The four diagnostic criteria for EAM were suggested by Sampson, who first reported the malignant transformation of endometriosis in 1925 (3), and were supplemented by Scott (4). They are as follows: (I) coexistence of benign and malignant lesions in the ipsilateral ovary, (II) presence of malignant lesions originating from the endometriosis area rather than by metastasis, (III) histological similarity to endometrial stroma and glandular epithelium, and (IV) morphological continuity of transition from benign to malignant tissue in endometriosis. All the criteria were satisfied in this case.

Patients with endometriosis are currently considered to be at higher risk of EAM under certain conditions, which include age ≥ 45 years, high levels of CA125, lesion of diameter ≥ 10 cm, and history of endometriosis ≥ 8 years. A study showed that high levels of CA125 are associated with invasion of endometriosis, which indicates a high risk of malignancy (5). The diameter of the lesion in our patient was more than 10 cm, with a tendency to increase even more. The larger the diameter of the lesions, the more likely the lesions are accompanied by tissue adhesions, and a higher degree of malignancy implies a greater difficulty of treatment (6, 7). In addition, excessive endogenous and exogenous estrogen due to aging, obesity, and estrogen replacement therapy after hysterectomy can also cause EAM (8, 9). This is because changes in hormone levels in the body can stimulate endometrial hyperplasia and increase the possibility of endometrial tissue migration or metastasis, which raises the risk of EAM (10). In our case, the patient had the following high-risk factors: advanced age (63 years), high levels of CA125 (258.32 U/ml), and rectal mass of large size (12 cm in diameter).

Until now, seven cases of clear cell adenocarcinoma have been reported to result from bowel endometriosis, as far as we know. The average age of the patients was 56.3 years, and the main complaint of all the patients was hematochezia (2). It was rare to report that rectal endometriosis can induce high-grade serous carcinoma and is complicated with ovarian cancer. In our case, the patient had concurrent primary carcinoma; and malignant transformation of rectal endometriosis that did not result from ovarian cancer metastasis as the transitional zone, observed on histopathological examination, was composed of abnormal cells and glands between the rectal tumor and normal healthy mucosa (see Figure 2A). There was no transition zone on the ovary. Additionally, we found no endometriosis lesions on the ovary during the operation, whereas, on the anterior rectal wall, we found a smooth, intact cyst. During the process of stripping the cyst, a thick brown liquid flowed out. This observation was consistent with the clinical features of endometriosis lesions. If it was result of the ovarian cancer metastasizing, it should belong to the advanced stage of ovarian cancer, when the lesion in the intestine should be a cauliflower-like tissue. The examination also revealed that the ER staining of endometrial glands and stroma near the rectal tumors was positive, Furthermore, immunohistochemical test of the rectal tumors presented positive for WT-1 staining and progesterone receptor (PR) staining. Therefore, this case was diagnosed as malignant transformation caused by rectal endometriosis.

In the clinical sense, the current inclusion criteria for EAM are not uniform. For example, some studies used patient self-reported histories of endometriosis, whereas other studies considered the criteria for cases of endometriosis with concurrent ovarian cancer. A few studies on endometriosis-associated ovarian carcinoma strictly met the Scott diagnostic criteria. Most of the cases were due to the low rate of surgical diagnosis of endometriosis, as many pathologists do not perform examinations for endometriosis lesions once ovarian cancer has been diagnosed. Fast-growing cancer cells may then replace the endometriosis lesions, thereby eliminating histological evidence of endometriosis. Our case had been misdiagnosed as ovarian carcinoma preoperatively.

It is difficult to diagnose EAM preoperatively because the medical history of a large number of carcinoma patients with a history of endometriosis is unclear. There is currently a lack of systematic meta-analysis of EAM samples, and there is no established screening method that is effective for risk prediction of EAM (9). The 5-year survival rate of patients with EAM is 77.7%. Because EAM is basically a low-grade or low-stage tumor, surgical resection is the first-choice treatment for patients with extragonadal EAM, especially when it occurs in the rectum or rectovaginal septum, and surgical resection is followed by radiotherapy (11). In addition to surgery, hormone therapy and systemic chemotherapy are other treatment modalities that have been reported in published literature (12). Our patient postoperatively received 6 cycles of chemotherapy consisting of carboplatin and paclitaxel and were followed up for 1 year with no recurrence of the condition. We will continue to follow up on the patient.

## Data Availability Statement

The datasets generated for this study are available on request to the corresponding author.

## Ethics Statement

This study was carried out in accordance with the recommendations of KYLL-2015-077, the Ethics Committee of Qilu Hospital of Shandong University with written informed consent from all subjects. All subjects gave written informed consent in accordance with the Declaration of Helsinki. Written consent was obtained, in accordance with the Declaration of Helsinki, from the patient for the analysis and publication of this case report and any accompanying images. The protocol was approved by the Ethics Committee of Qilu Hospital of Shandong University.

## Author Contributions

GW read and approved the final manuscript. LS performed the data analyses and wrote the manuscript. AX performed the pathological analysis. QL helped perform the analysis with constructive discussions.

### Conflict of Interest

The authors declare that the research was conducted in the absence of any commercial or financial relationships that could be construed as a potential conflict of interest.
